# Early effect of a single intravenous injection of ethanol on hepatic sinusoidal endothelial fenestrae in rabbits

**DOI:** 10.1186/1476-5926-8-4

**Published:** 2009-07-13

**Authors:** Frank Jacobs, Eddie Wisse, Bart De Geest

**Affiliations:** 1 Center for Molecular and Vascular Biology, Department of Molecular and Cellular Medicine, University of Leuven, Herestraat 49, Leuven, 3000, Belgium; 2 EM Unit Pathology Department, University of Maastricht, Universiteitssingel 50, Maastricht, 6229-ER, the Netherlands; 3 Department of Internal Medicine, University of Maastricht, Universiteitssingel 50, Maastricht, 6229-ER, the Netherlands

## Abstract

**Background:**

It has been postulated that ethanol affects hepatic sinusoidal and perisinusoidal cells. In the current experimental study, we investigated the early effect of a single intravenous dose of ethanol on the diameter of liver sinusoidal endothelial fenestrae in New Zealand White rabbits. The diameter of fenestrae in these rabbits is similar to the diameter found in humans with healthy livers. The effect of ethanol on the size of fenestrae was studied using transmission electron microscopy, because plastic embedding provides true measures for the diameter of fenestrae.

**Results:**

After intravenous administration of a single dose of 0.75 g/kg, ethanol concentration peaked at 1.1 ± 0.10 g/l at ten minutes after injection. Compared to control rabbits (103 ± 1.1 nm; n = 8), the average diameter of fenestrae in ethanol-injected rabbits determined at 10 minutes after injection was significantly (p < 0.01) smaller (96 ± 2.2 nm; n = 5). Detailed analysis of distribution histograms of the diameters of fenestrae showed that the effect of ethanol was highly homogeneous.

**Conclusion:**

A decrease of the diameter of fenestrae 10 minutes after ethanol administration is likely the earliest morphological alteration induced by ethanol in the liver and underscores the potential role of liver sinusoidal endothelial cells in alcoholic liver injury.

## Background

It has been postulated that ethanol primarily targets hepatic sinusoidal and perisinusoidal cells [1]. In experimental models and in human studies, plasma hyaluronic acid levels are elevated in alcoholic liver injury, which may reflect a diminished hepatic clearance by liver sinusoidal endothelial cells [2-4]. Chronic ethanol exposure leads to defenestration in liver sinusoidal endothelial cells which is paralleled by the deposition of a basal lamina [5]. Subsequently, capillarization of hepatic sinusoids further impairs microcirculatory exchange of nutrients and the clearance of waste products, enhances tissue fibrosis, and will affect the hepatic parenchyma and its metabolism. Whereas this sequence of events has been corroborated by several studies, it is not well established to which extent a single administration of ethanol affects liver sinusoidal endothelial cells. Previous studies have shown that ethanol slightly (6%) increases the diameter of fenestrae in liver sinusoidal endothelial cells *in vitro *[6,7]. In contrast, scanning electron microscopy studies *in vivo *showed significant decreases of the diameter of sinusoidal endothelial fenestrae [8], suggesting that the transport of plasma substances from sinusoids to parenchymal liver cells may already be impaired by acute ethanol intake. Because scanning electron microscopy is applied on dried and thus shrunken specimens, *lege artis *determination of the diameter of fenestrae requires transmission electron microscopy of plastic-embedded specimens. Quantification of the diameters in these sections is performed on fenestrae that become visible as holes when the sinusoidal wall is cut tangentially. The goal of the current investigation was to establish unambiguously whether a single intravenous injection of ethanol administration has an effect on the diameter of fenestrae *in vivo*. We have recently shown that the diameter of fenestrae in human healthy livers, fixed by injecting glutaraldehyde into fresh wedge biopsies, is similar compared to fenestrae in the livers of New Zealand White rabbits [9] and is significantly smaller than in mice [10] or rats [11]. Therefore, diameters were determined using transmission electron microscopy ten minutes after injection of ethanol or 0.9% NaCl in New Zealand White rabbits.

## Results

**Figure 1:**
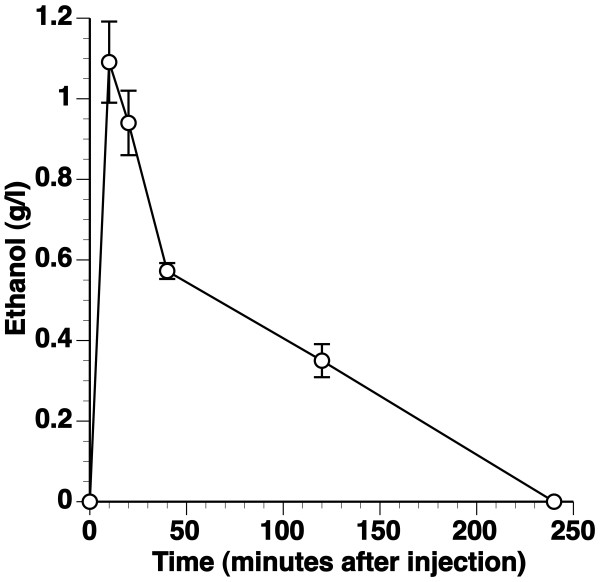
**Plasma ethanol concentrations in New Zealand White rabbits**. Ethanol concentration (g/l) in New Zealand White rabbits injected with 0.75 g/kg ethanol. Data are expressed as means ± SEM (n = 5).

A dose of 0.75 g/kg ethanol was administered intravenously via a marginal ear vein to male New Zealand White rabbits. The ethanol concentration in plasma is shown in Figure 1. Ethanol concentration peaked at 1.1 ± 0.10 g/l (n = 5) at 10 minutes and was 0.35 ± 0.041 g/l (n = 5) at 2 hours after injection. Ethanol was below detection limit (0.06 g/l) at 4 hours after injection. The time-point corresponding to the peak ethanol concentration (10 minutes after injection) was chosen to determine the diameter of fenestrae by transmission electron microscopy.

**Figure 2:**
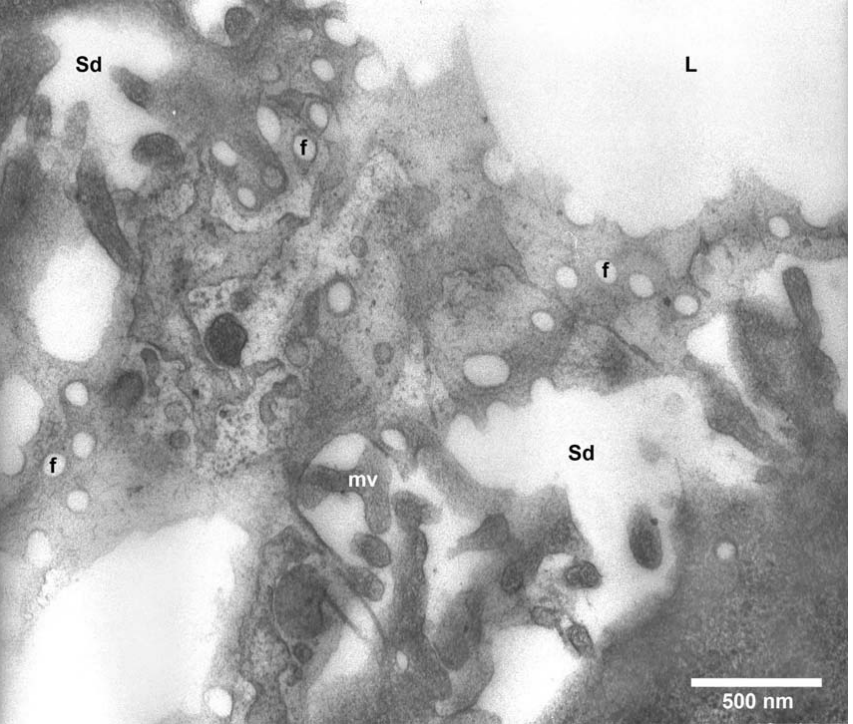
**Transmission electron micrograph of liver sinusoidal endothelial fenestrae in New Zealand White rabbits**. The endothelial lining is cut tangentially and shows the occurrence of fenestrae (f) mostly in groups, called sieve plates. To the left and the right hand side of the picture, we find the space of Disse (Sd) with sparse microvilli (mv) protruding from parenchymal cells. The right top corner of the picture shows the lumen (L) of the sinusoid. The right bottom part of the picture shows the cytoplasm of a parenchymal cell.

**Figure 3:**
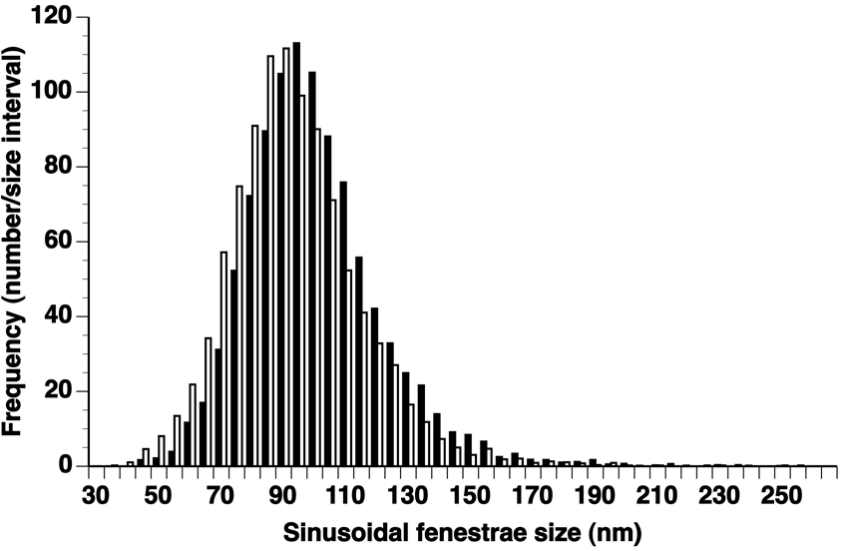
**Frequency distribution histograms of the diameter of liver sinusoidal endothelial fenestrae in New Zealand White rabbits**. Comparison of the frequency distribution histograms of the size of sinusoidal fenestrae in New Zealand White control rabbits (black bars; n = 8) and New Zealand White rabbits injected with 0.75 g/kg ethanol 10 minutes before perfusion fixation (white bars; n = 5). Each bar corresponds to a 5 nm interval.

A representative transmission electron micrograph used to measure the diameter of fenestrae in male New Zealand White rabbits is shown in Figure 2. The average number of measurements per liver was 640 ± 98 (n = 8) and 690 ± 67 (n = 5) in 0.9% NaCl and ethanol-injected rabbits, respectively. The frequency distribution histogram of diameters of liver sinusoidal fenestrae determined by transmission electron microscopy 10 minutes after injection of 0.9% NaCl or ethanol is provided in Figure 3. Compared to control rabbits (103 ± 1.1 nm), the average diameter of fenestrae in ethanol-injected rabbits was significantly smaller (96 ± 2.2 nm; p < 0.01). The effect of ethanol on the diameter of fenestrae was homogeneous (Figure 3) as evidenced by significant reductions of the percentile 10 (72 ± 1.7 nm *versus *79 ± 1.1 nm; p < 0.01), percentile 25 (82 ± 1.7 nm *versus *89 ± 1.3 nm; p < 0.05), median (94 ± 1.8 nm *versus *100 ± 1.3 nm; p < 0.05), percentile 75 (107 ± 2.3 nm *versus *113 ± 1.4 nm; p < 0.05) and percentile 90 values (122 ± 3.2 nm *versus *130 ± 1.4; p < 0.05 nm) in ethanol-treated rabbits compared to control rabbits.

## Discussion

The current study, using *lege artis *transmission electron microscopy measurements, shows that ethanol at toxicologically relevant levels significantly decreases the diameter of fenestrae in New Zealand White rabbits. Since this effect was observed ten minutes after ethanol injection, this study is in line with the view that the liver sinusoidal endothelial cells are the first hepatic cells that undergo morphological changes in alcoholemia [1].

Both endothelin-1 and NO may play a role in the effect of ethanol on the diameter of fenestrae. Previously, it has been demonstrated that ethanol induces hepatic vasoconstriction in isolated perfused rat liver and that endothelin-1 antibodies significantly inhibit this ethanol-induced hepatic vasoconstriction [12]. Since endothelin-1 has been shown to induce contraction of hepatic sinusoidal endothelial fenestrae [13], endothelin-1 may mediate the decrease of the diameter of fenestrae after ethanol injection. Although hepatic vasoconstriction in isolated perfused rat liver persists during ethanol exposure, portal pressure gradually decreases [12]. This attenuation of ethanol induced vasoconstriction is mediated by NO[12]. Similarly, NO may oppose the contraction of hepatic sinusoidal endothelial fenestrae by endothelin-1: it induces a decrease in the cytosolic free calcium concentration leading to the dissociation of calcium and calmodulin from the myosin light chain kinase. Under these conditions, myosin light chain phosphatase dephosphorylates the myosin light chain and causes relaxation of fenestrae [14]. NO bioavailability in the sinusoid in the presence of ethanol will depend on two opposing factors. On the one hand, ethanol increases both endothelial NOS (eNOS) expression and NO production [15]. On the other hand, ethanol has also been shown to induce a release of superoxide anions into the hepatic sinusoid [16,17], reducing NO bioavailability. The source of superoxide may be the liver sinusoidal endothelial cells [16] themselves as well as Kupffer cells [17]. Differences in endothelin-1 production and NO bioavailability between the *in vitro *setting and *in vivo *experiments may explain the discrepant results between different studies [6-8]. Whereas previous *in vitro *studies [6,7] have shown that ethanol slightly increases the diameter of fenestrae in liver sinusoidal endothelial cells, an *in vivo *scanning electron microscopy study in rats showed significant decreases in the diameter of sinusoidal endothelial fenestrae [8], similar as in the current study.

Previously, it has been shown that acute ethanol administration in Balb/c mice increased hyaluronic acid levels, a functional marker for sinusoidal endothelial liver cells, at 3 hours and 6 hours, whereas alanine aminotransferase levels, a marker of hepatocyte damage, were unchanged [4]. In the current study, a decrease of the diameter of fenestrae was observed as early as 10 minutes after injection. This may be the first effect of ethanol on liver sinusoidal endothelial cells and the earliest morphological alteration induced by ethanol in the liver. The smaller diameter of sinusoidal endothelial fenestrae following acute ethanol intake may induce a decrease of microcirculatory exchanges between the sinusoidal lumen and the space of Disse. This may contribute to protection of parenchymal liver cells from the toxic effects of ethanol.

## Conclusion

The current study, showing a reduced diameter of fenestrae within 10 minutes following a single intravenous ethanol administration, underscores the potential role of liver sinusoidal endothelial cells in alcoholic liver injury. The reduction in the diameter of sinusoidal fenestrae may reduce the exchange between the sinusoidal lumen and the space of Disse and may therefore contribute to protecting parenchymal liver cells from the toxic effects of ethanol.

## Methods

### Animal experiments

All experimental procedures in animals were performed in accordance with protocols approved by the Institutional Animal Care and Research Advisory Committee. The investigation conforms with the *Guide for the Care and Use of Laboratory Animals *published by the US National Institutes of Health (NIH Publication No. 85-23, revised 1996). New Zealand White rabbits were obtained from the University of Gent (Merelbeke, Belgium). Experiments were performed at the age of 4 months.

### Study design

A dose of 0.75 g/kg ethanol was administered intravenously via a marginal ear vein to male New Zealand White rabbits (n = 5) at the age of 3 months and blood sampling was performed at 0 minutes, 10 minutes, 30 minutes, 2 hours and 4 hours. In separate experiments, male New Zealand White rabbits were intravenously injected with 0.9% NaCl (n = 8) or 0.75 g/kg ethanol (n = 5) 10 minutes before perfusion fixation of the rabbit liver. The average weight of rabbits in these experiments was 2.9 ± 0.25 kg (n = 18) and was not significantly different between different groups.

### Blood sampling

Blood was obtained from the central ear artery and anticoagulated with 1/10 volume of trisodium citrate. Samples were taken after an overnight fast.

### Determination of ethanol concentrations in plasma

Plasma ethanol concentrations were measured using the alcohol dehydrogenase assay-based ethyl alcohol Flex™ reagent cartridge (Dade Behring Inc., Newark, DE, U.S.A.) on a Dade Behring Dimension^® ^automated clinical chemistry analyzer (Dade Behring Inc.).

### Quantification of the size of sinusoidal fenestrae by transmission electron microscopy

Perfusion of the rabbit liver with a fixative solution was performed essentially as described before [18-20]. After isoflurane anesthesia and exposure of the liver by laparotomy, the hepatic artery and common bile duct were clamped and two ligatures were placed around the portal vein. A sharpened 14-gauge pipette was introduced in the portal vein and fixed by tightening the two ligatures. Perfusion fixation was performed at a pressure of 15 cm H_2_O with 250 to 300 ml of 1.5% glutaraldehyde fixative buffered in 0.067 M cacodylate at pH 7.4. The inferior caval vein was transsected at the start of the perfusion. The perfusion was continued until the colour of the liver changed from dark reddish brown to yellow brown and the consistency from soft to stiff (equivalent to the stiffness of a hard boiled egg). The liver was removed and thin slices were cut with a razor blade into 30–40 1 mm^3 ^blocks from a left liver lobe as well as from a right liver lobe. These blocks were washed in cacodylate buffer and transferred to a 1% OsO_4 _fixative solution buffered with phosphate buffered saline 0.1 M pH 7.4 for subsequent immersion fixation during 1 hour at 4°C. After washing in phosphate buffered saline 0.1 M pH 7.4, dehydration was carried out rapidly in graded ethanol series (70°–100°), followed by embedding in Epon. Sections with a thickness of 2 μm were cut for light microscopy to check the quality of the fixation and embedding. Subsequently, ultrathin sections for transmission electron microscopy were cut with an ultramicrotome with diamond knife. These sections have a typical thickness of 60 nm. Five to ten ultrathin sections with a length and width of 500 to 1000 μm were mounted on 75 mesh copper grids (3 mm diameter) with a carbon-coated Formvar film, and subsequently contrasted with uranyl acetate and lead citrate. As a size reference, a calibration grid with a spacing of 463 nm was photographed at a magnification of 8400 × at the beginning of each session. The specimens were examined at the University of Maastricht (EM unit, Pathology) in a Philips CM 100 (F.E.I., Eindhoven, The Netherlands) at 80 kV. The size of fenestrae was measured as the largest diameter in sections that cut the endothelial wall tangentially and show the fenestrae as complete holes. Measurements were performed manually on a monitor using ImageJ software (Wayne Rasband, National Institutes of Health, USA, ). For each rabbit, ultrathin sections originating from two independent 1 mm^3 ^blocks (corresponding to the right and left liver lobe) were analysed.

### Statistical analysis

All data are expressed as means ± standard error of the means (SEM). The diameters of fenestrae in saline and ethanol-injected rabbits were compared by a Student's t-test using Instat3 (GraphPad Software). Gaussian distribution of the data was tested using the method of Kolmogorov and Smirnov. The homogeneity of variances between groups was checked with Levene's test for equality of variances. A two-sided p-value of less than 0.05 was considered statistically significant.

## Competing interests

The authors declare that they have no competing interests.

## Authors' contributions

FJ and EW acquired, analysed and interpreted data. EW and BDG conceived and designed the research. All authors made critical revision of the manuscript for important intellectual content.
